# Automated Measurement of Heart Girth for Pigs Using Two Kinect Depth Sensors

**DOI:** 10.3390/s20143848

**Published:** 2020-07-10

**Authors:** Xinyue Zhang, Gang Liu, Ling Jing, Siyao Chen

**Affiliations:** 1 Key Laboratory of Modern Precision Agriculture System Integration Research, Ministry of Education, China Agricultural University, Beijing 100083, China; s20183081347@cau.edu.cn; 2 Key Laboratory of Agricultural Information Acquisition Technology, Ministry of Agriculture, and Rural Affairs, China Agricultural University, Beijing 100083, China; 3 College of Science, China Agricultural University, Beijing 100083, China; jingling@cau.edu.cn; 4 Graduate School of Agriculture, Kyoto University, Kyoto 606-8502, Japan; chen.siyao.86z@st.kyoto-u.ac.jp

**Keywords:** pig, point cloud registration, heart girth, Kinect, automatic measurement

## Abstract

The heart girth parameter is an important indicator reflecting the growth and development of pigs that provides critical guidance for the optimization of healthy pig breeding. To overcome the heavy workloads and poor adaptability of traditional measurement methods currently used in pig breeding, this paper proposes an automated pig heart girth measurement method using two Kinect depth sensors. First, a two-view pig depth image acquisition platform is established for data collection; the two-view point clouds after preprocessing are registered and fused by feature-based improved 4-Point Congruent Set (4PCS) method. Second, the fused point cloud is pose-normalized, and the axillary contour is used to automatically extract the heart girth measurement point. Finally, this point is taken as the starting point to intercept the circumferential perpendicular to the ground from the pig point cloud, and the complete heart girth point cloud is obtained by mirror symmetry. The heart girth is measured along this point cloud using the shortest path method. Using the proposed method, experiments were conducted on two-view data from 26 live pigs. The results showed that the heart girth measurement absolute errors were all less than 4.19 cm, and the average relative error was 2.14%, which indicating a high accuracy and efficiency of this method.

## 1. Introduction

The body size parameters of pigs are important indices for healthy pig breeding [1]. They not only directly reflect the growth and development of pigs, but also are closely related to production performance, health status and meat quality [2,3]. Simple linear calibration equation using heart girth and body length as independent variables can also be used to predict the body weight of pigs [4]. Therefore, the accurate measurement of pig size parameters is important for optimizing pig breeding and management [5]. The traditional method of measuring livestock body size is via manual tape measurement. On the one hand, this method is easily affected by the subjectivity of the person performing the measurement and external environmental factors, and it is not suitable for large-scale breeding operations [6]. On the other hand, contact measurements can produce a stress response in livestock, affect their growth and development, and reduce animal health and welfare [7]. As an alternative, non-contact body measurements of livestock through machine vision technology can effectively overcome the disadvantages of traditional measurement methods and improve measurement efficiency [8].

Machine vision technology for measuring the size of livestock has advanced from methods based on two-dimensional images to methods using three-dimensional point clouds [9,10,11]. Methods of using two-dimensional images to measure the body size of livestock are easy and inexpensive [12,13], but they can be affected by complex background and lighting conditions, and only limited body size information can be collected [14]. To overcome the difficulty of extracting livestock body size parameters from two-dimensional images, Chen et al. [15] used a binocular vision system to obtain a three-dimensional point cloud of a pig to calculate the body size; however, this binocular vision system still suffered from the influence of lighting variations. With the emergence of consumer-level depth sensors such as the ASUS Xtion PRO sensor (ASUS TeK Computer Inc., Taipei, Taiwan) and the Microsoft Kinect sensor (Microsoft Corporation, China), a new avenue for acquiring 3D point clouds of livestock has become available [16,17]. ASUS Xtion PRO sensors (ASUS TeK Computer Inc., Taipei, Taiwan) were used by Guo et al. [18] to collect pig point clouds and realize interactive body size measurements. Salau et al. [19] used a depth sensor based on the time-of-flight (TOF) principle to collect a local point cloud of a cow’s buttocks and verified the feasibility of using such a depth sensor for the measurement of livestock body size. Single-view depth image acquisition is simple and does not require data registration; however, this approach supports only linear body measurements, such as the length, width, and height, and cannot be used for the accurate measurement of heart girth, hip circumference or other circumferential body size parameters [20,21,22]. The use of multi-view depth sensors to measure livestock body size [23,24,25,26,27] has also been reported. Pezzuolo et al. [28] used multiple Kinect sensors (Microsoft Corporation, Beijing, China) to obtain local point cloud data for cows from different angles at close range and measured the head size, hip distance, withers-to-tail length, chest girth, hip circumference, and withers height of the cows using the commercial software SPIP. To realize the non-contact automated measurement of a pig’s heart girth, Wang et al. used two ASUS Xtion PRO sensors (ASUS TeK Computer Inc. Taipei, Taiwan) to collect point cloud data from above the sides of the pig’s body and applied the b-spline curve fitting method [29]. However, due to the influence of the sensors’ angles of view, data on the pig’s abdomen dimensions were missing. Hence, the results obtained through curve fitting were somewhat different in shape from the actual heart girth profile. Therefore, the accuracy of heart girth measurement depends largely on the fitting model.

The aim of study is to improve the accuracy of the measurement of the heart girth of pigs. To achieve this aim, this paper proposes a method of automated heart girth measurement based on two Kinect depth sensors synchronously acquiring depth images of a pig from the top and side views. Using the proposed data acquisition platform, complete data from the back and one side of the pig’s body (including data from one side of the abdomen) are collected. Using mirror symmetry, a complete point cloud of the heart girth region is obtained, thus providing a basis for higher-precision heart girth measurements. Our contributions can be summarized as follows:

(1) We built a two-view pig depth image acquisition platform. The 4PCS method is improved according to the physical characteristics of the pig and the geometric characteristics of the two-view area to realize the registration of the top- and side-view pig point clouds.
(2) In the process of measurement, a method for automatically extracting heart girth measurement point and a method measures pig heart girth along the point cloud using the shortest path method are proposed.

## 2. Materials and Methods

### 2.1. Platform Construction and Data Acquisition

**Figure 1:**
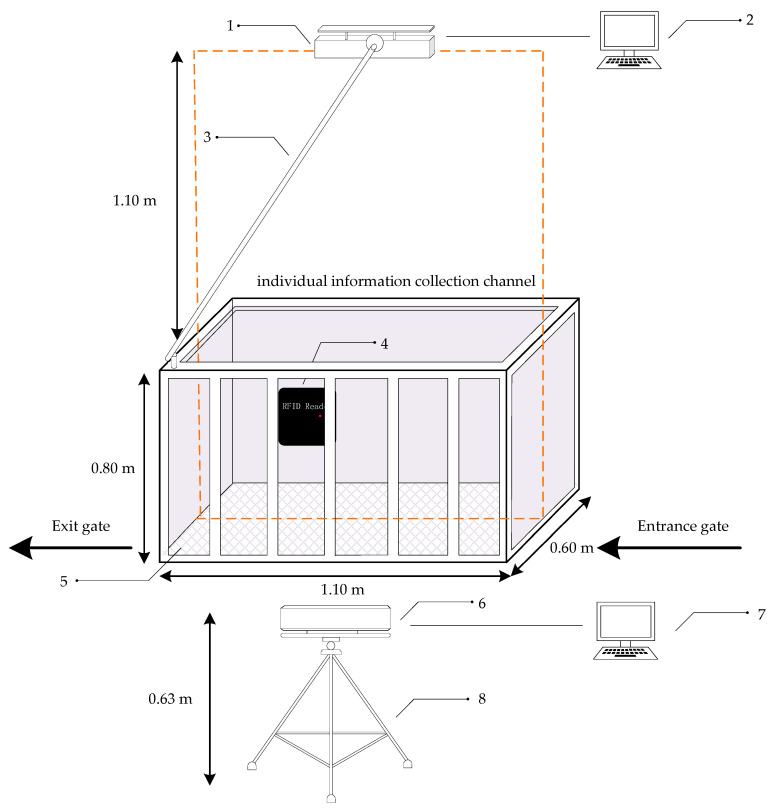
Data acquisition platform constructed in the information collection channel: **1**—top-view Kinect; **2**—computer connected to the top-view Kinect; **3**—rocker; **4**—RFID reader; **5**—weight scale; **6**—side-view Kinect; **7**—computer connected to the side-view Kinect; **8**—tripod.

In October 2019, we used two Kinect depth sensors (Microsoft Corporation, Beijing, China) to acquire depth images of 26 pigs at the Tianpeng Xingwang Farm in Beijing. These pigs were two breeds, pure Large White and pure Landrace. At the experimental pig house, there is an automatic feeding system for the pigs. Pigs enter the automatic feeding system for feeding every day following their own feeding routines. The whole feeding system is composed of two parts: an individual information collection channel and a feeding area. After a pig enters the feeding system, it is weighed in the individual information collection channel and then proceeds to the feeding area. The individual information collection channel is approximately 1.10 m long, 0.60 m wide and 0.80 m high. Only one pig can pass through this channel at a time. A data acquisition platform was built on the basis of this channel, as shown in Figure 1. The experimental platform included an RFID reader, two Kinect V2 sensors (Microsoft Corporation, Beijing, China) and two computers connected to and controlling the platform.

Since the effective sight distance of the Kinect V2 is 0.5–4.5 m, to capture multiple complete pig depth images and reduce the impact of environmental factors on the Kinect sensors (Microsoft Corporation, Beijing, China), the Kinect sensor used to collect the top-view images was placed approximately 1.10 m above the channel based on the results of preliminary tests, and this Kinect sensor was aligned with the central axis of the channel. The Kinect sensor for collecting side-view images was placed at a distance of 0.63 m from the ground and approximately 0.94 m from the channel fence. With these dimensions, the best data acquisition effect was achieved.

After a pig entered the channel, the entrance and exit gates were closed to ensure that there would be only one pig in the channel. After the current pig’s ear tag was acquired by the RFID reader, the two Kinect sensors (resolution: 512 × 424 pixels) simultaneously began to collect top-view and side-view depth images, with a frame rate of 30 frames per second. Each collected depth image was associated with a time stamp to enable the frame synchronization of the pig images acquired from the two views in order to facilitate subsequent pig point cloud registration.

### 2.2. Data Preprocessing

**Figure 2:**
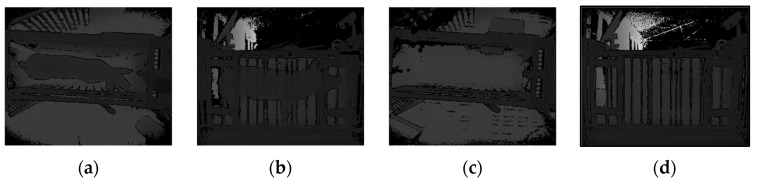
Original depth images: (**a**) Top-view pig depth image; (**b**) side-view pig depth image; (**c**) Top-view depth image of the individual information collection channel acquired with no pig in the channel; (**d**) Side-view depth image of the individual information collection channel acquired with no pig in the channel.

**Figure 3:**
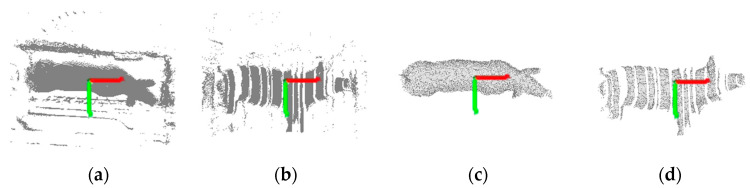
Pig point cloud preprocessing. In these images, the red axis is the *x*-axis, and the green axis is the *y*-axis. (**a**) and (**b**) Top- and side-view pig point clouds extracted from depth images after background difference processing; (**c**) and (**d**) Top- and side-view pig point clouds obtained after filtering and segmentation.

Pig depth images collected simultaneously from the top and side views are shown in Figure 2a,b. Due to the complex environment of the farm, in addition to the pig data, the original data collected by the Kinect sensors include data from complex background objects such as the weight scale, railings and ground. Consequently, it was necessary to extract the pig data from this complex background. The specific steps of the extraction process are as follows:

(1) Background images (Figure 2c,d) acquired from the top and side views were used as masks, and the background difference method [30] was applied to extract the foreground of each collected image to isolate the pig depth image and convert it into a point cloud, as shown in Figure 3a,b.
(2) Pass-through filtering was used to filter out non-interesting background points from the original pig body point cloud.
(3) Point cloud downsampling can reduce redundant data and improve measurement efficiency. Voxel grid filtering was used to downsampling of the pig point cloud.
(4) The neighbourhood average distance threshold method was used to dynamically remove outliers from the pig body surface.
(5) Euclidean clustering segmentation [31] was used to segment the point cloud. The cluster with the largest number of points in the clustering results was taken as the pig point cloud, as shown in Figure 3c,d.
(6) Due to occlusion by the railings, some parts of each pig point cloud were missing, producing nonrigid deformations. The CloudCompare software was used to remove the corresponding deformed boundary data for subsequent registration and measurement.

### 2.3. Registration Two-View Pig Point Clouds with Feature-Based Improved 4PCS Method

**Figure 4:**
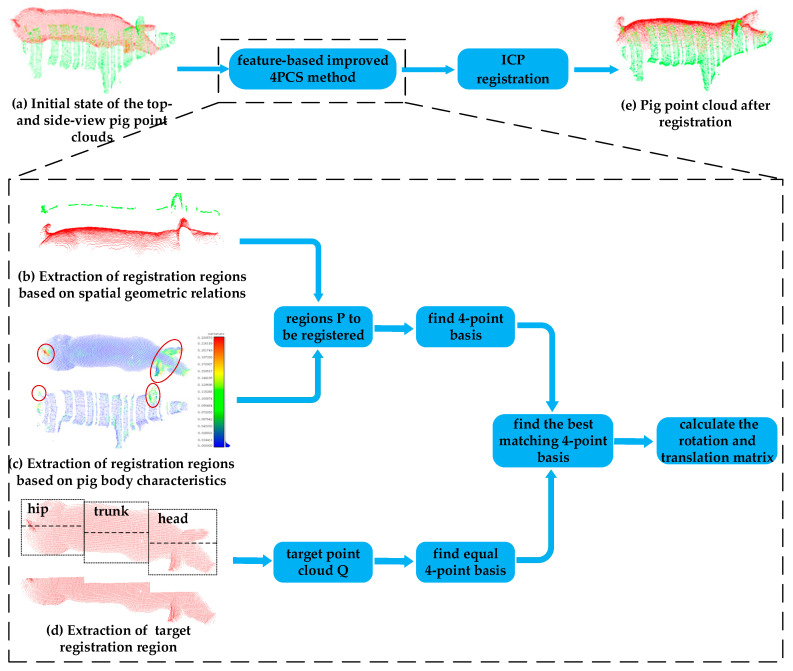
Illustration of the registration process for two-view pig point clouds. The top-view point cloud is shown in red, and the side-view point cloud is shown in green. (**a**): Initial state of the top- and side-view pig point clouds; (**b**): Extraction of registration regions based on spatial geometric relations; (**c**): Extraction of registration regions based on pig body characteristics; (**d**): Extraction of target registration region; (**e**): Pig point cloud after registration.

The top-view point cloud and side-view point cloud collected synchronously (as shown in Figure 4a) are two independent point clouds in different coordinate system. The top-view point cloud mainly reflects the information of the pig’s back, while the side-view point cloud mainly reflects the information of one side of the pig’s body, so it is impossible to directly measure the pig heart girth. Therefore, we need to perform the registration of two point clouds to form a complete pig point cloud and provide conditions for subsequent pig heart girth measurement.

At present, the most widely used point cloud registration method is the iterative closest point (ICP) algorithm [32,33], which requires only simple calculations and offers high registration accuracy. However, the registration accuracy depends on the initial relative pose of the two input point clouds. Furthermore, the ICP algorithm requires sufficient overlap between the two point clouds to be registered. Therefore, for point cloud registration, coarse registration of the two-view point clouds to be registered is usually performed first, and then the ICP algorithm is used for fine registration. For the pig point clouds of interest in this study, there are large differences in position and pose between the top- and side-view point clouds of the same pig, and the coincidence rate between points in the two point clouds is low. If the 4-Point Congruent Set (4PCS) method [34,35] of coarse registration were to be used directly, registration would easily fail. Therefore, the regions of the two point clouds that are to be registered must first be extracted by considering the body features of a pig; specifically, regions with rich information in the form of boundary features as well as ear and tail features are used as the registration regions. Thus, the performance of the 4PCS algorithm is improved, and the ICP algorithm can subsequently be used to achieve the registration of the two-view pig point clouds, as shown in Figure 4.

#### 2.3.1. Extraction of Registration Regions from Two-View Pig Point Clouds

The difference between the initial positions and poses of the pig point clouds collected from the top view and the side view is large, and the overlap rate of these point clouds is low, as shown in Figure 4a. The side-view point cloud is partially missing due to barrier occlusion, and the edges of the missing regions show some nonrigid deformations, which increase the difficulty of registration. If the registration of the two-view point clouds were to be carried out directly, there would be too many invalid registration pairs, resulting in low registration accuracy or even failure. Due to the influence of the sensor angles and the pig’s own characteristics, it is impossible to directly locate the overlapping regions. From an analysis of spatial geometry, it can be seen that the upper part of the side-view contour of any object exists and is unique in its top-view contour [36]. Thus, there is a unique correspondence between the back contour of the side-view pig point cloud and the top-view pig point cloud, as shown in Figure 4b; therefore, the contour of the pig’s back from the side-view point cloud is used as one of the regions P to be registered. The pig contour is extracted, and the details of the contour extraction method are shown in the Appendix A. Take the maximum and minimum values of the pig contour in the *y*-axis direction, *y_max_* and *y_min_*, and extract the point cloud consisting of $y\in \{y_{min},y_{min}+(y_{max}-y_{min})/4\}$ as the back profile of the pig from the side-view point cloud.

It is very important to find corresponding point pairs in point cloud registration process, which directly affects the accuracy of point cloud registration. The larger the curvature change, the richer the feature information is, the easier to match the corresponding points accurately [37]. The principal component analysis (PCA) [38] method is used to calculate the curvature at each point in the top- and side-view point clouds, as shown in Figure 4c. It can be seen from this figure that the back curvature of a pig is relatively uniform, the curvature change is small, the associated feature information is relatively weak. If only the back information were to be used for registration, the corresponding points were easy to match wrong, leading to low registration accuracy and even registration failure. By contrast, the curvature values of the ears and tail of a pig are significantly larger than those of the back, and the curvature changes obviously, providing more characteristic information, which is helpful to accurately find the corresponding point and improve the registration accuracy. We rank the unordered side-view points of the pigs in order of curvature value from large to small. Because the curvature values in the ear and tail regions are larger than those in other regions, we can extract the ear and tail regions by extracting the first M (set M = 500) points ordered in this way from the side-view pig point cloud. The extracted ear and tail regions of the side-view pig point cloud are then combined with the back contour from the side-view pig point cloud as the region P to be registered.

If we directly regard the top-view point cloud as the target point cloud Q, when the pig’s posture is good and the trunk is relatively straight, the point cloud acquired from the right side of the body can be easily registered with the left. Therefore, we divide the top-view pig point cloud into left and right parts along the central axis of the body and take only the side corresponding to the side-view point cloud as the target point cloud Q to reduce registration errors. Considering that there is often slight bending or turning of the pig’s body, the point cloud representing the pig’s back is divided into three parts: hip, trunk and head, as shown in Figure 4d. The central axis of each part is identified individually. Then this axis is used to extract this part of top-view point cloud which corresponds with the side-view point cloud. 

#### 2.3.2. Registration of Two-View Pig Point Clouds

The 4PCS algorithm for coarse registration searches for a rigid transformation based on the minimum distance between two point clouds by constructing and matching a full 4-point basis between the two point clouds. Extracting specific registration regions from two-view pig point clouds as described in Section 2.3.1 can eliminate redundancies in this 4-point basis to some extent, thereby improving the performance of the 4PCS algorithm and reducing the necessary number of iterations of the algorithm.

(a) Select a point a in the region P to be registered. Calculate the maximum variable length *m* based on the diagonal length of the bounding box and the approximate overlap ratio of the region P to be registered. Then, select a second point *b* in the region P to be registered based on *m*, such that *a* and *b* satisfy:
  (1) ‖a−b‖=m
(b) Each scale factor should be affine invariant during point cloud translation and rotation. Calculate the scale factors *r*_1_ and *r*_2_ for the 4-point basis *B* = {*a,b,c,d*}:
  (2) $$\{\begin{array}{c} r_{1}=\frac{\left\|a-e\right\|}{\left\|a-b\right\|}\\ r_{2}=\frac{\left\|c-e\right\|}{\left\|c-d\right\|} \end{array},$$
  where *e* is the intersection of the vector *ab* and the vector *cd*.
(c) Repeat steps (a) and (b) to find all coplanar sets of four-point bases *E* = {*B*_1_, *B*_2_, …, *B_m_*} in the region P to be registered.
(d) Calculate the midpoints *e*_1_ and *e*_2_ of all pairs of points *q*_1_ and *q*_2_ in the target point cloud Q. If there are two pairs of such points in Q such that one pair with midpoint *e*_1_ and the other pair with midpoint *e*_2_ are equal within the allowable range of errors, then these two pairs of points can be considered to be corresponding coplanar points of the basis given in P.
  (3) $$\{\begin{array}{c} e_{1}=q_{1}+r_{1}(q_{2}-q_{1})\\ e_{2}=q_{2}+r_{2}(q_{2}-q_{1}) \end{array}$$

Traverse the target point cloud Q to find the set *D* = {*C*_1_, *C*_2_, …, *C_n_*} of all equal 4-point bases in Q. The largest common pointset (LCP) strategy [39] is used to find the best 4-point matching in the set D of the equal 4-point basis of equivalence, that is, to calculate the rotation and translation matrix of the point cloud, so as to achieve coarse registration of the two-view pig point clouds. Finally, the ICP algorithm is used to accurately register the two-view point clouds to achieve pig point cloud registration, as shown in Figure 4e.

### 2.4. Pig Heart Girth Measurement

The heart girth of a pig is the vertical circumference of the chest as measured with a tape along the posterior border of the scapula [40]. Manual measurement is typically performed using a tape measure wrapped around the pig’s chest in the axillary region behind the forelimbs to obtain this circumferential measurement. In a pig point cloud, the heart girth point cloud can be obtained by extracting a suitable heart girth measurement point to use as a starting point to intercept the circumferential perpendicular to the ground from the pig point cloud. In this paper, based on the contour features of the axillary region of the pig, a method of automatically extracting the heart girth measurement point is presented, and a shortest path method is proposed for measuring the distance along the contour of the corresponding heart girth point cloud.

#### 2.4.1. Heart Girth Measurement Point Extraction

**Figure 5:**
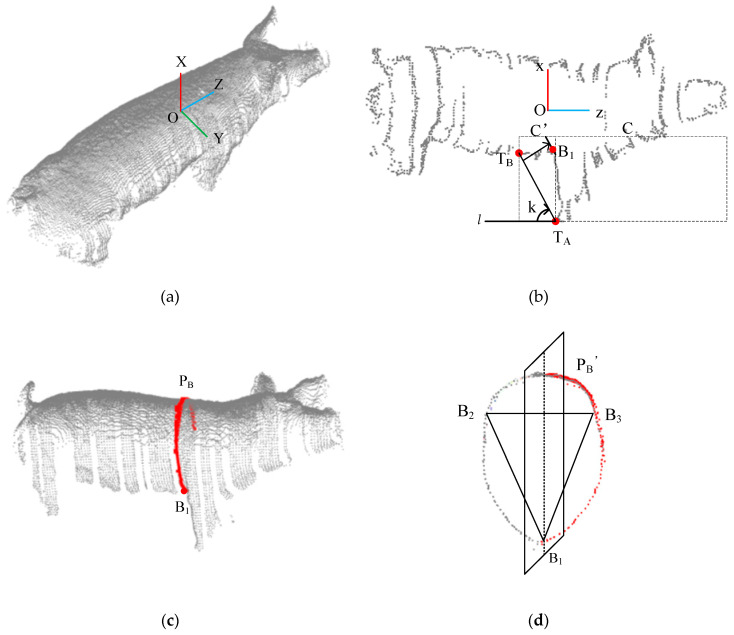
Heart girth measurement for a pig: (**a**) Pig point cloud after pose normalization; (**b**) Detection of the heart girth measurement point on the contour of the side-view point cloud; (**c**) The heart girth point cloud (red), which is obtained by taking the heart girth measurement point as the starting point and intercepting the circumferential perpendicular to the ground from the point cloud; (**d**) Application of mirror symmetry to obtain the whole heart girth point cloud, where the red points are those added to the point cloud through symmetrical filling.

After the method introduced in Section 2.3 is applied to register the two-view pig point clouds, the combined registered pig point cloud is further subjected to pose normalization [41]. The centre of gravity of the pig point cloud is taken as the origin O of the coordinate system. The direction pointing vertically upwards from the ground is the positive *x*-axis direction. The direction along the central axis of the pig’s back, pointing towards the head, is the positive direction of the *z*-axis. The axis oriented along the width direction of the pig’s back is the *y*-axis, and its positive direction is determined by the right-hand rule, as shown in Figure 5a. The registered pig point cloud is projected onto the XOZ surface, and the pig contour is extracted using the method described in Appendix A, as shown in Figure 5b. From the side-view point cloud contour, the concave point behind the forelimb is taken as the heart girth measurement point.

Pass-through filtering is then applied to obtain the local pig body point cloud C. The minimum point in the *x*-axis direction of the point cloud C is found to identify the front hoof point *T_A_* of the pig. Then, an envelope line *l*: x = *k*(*z*–*T_A_*), *k* ∈ (−∞, 0) is constructed through the front hoof point *T_A_*. Initially, the envelope line is oriented horizontally. Each point in the point cloud is traversed to judge the position relationship between that point and the envelope line in order to identify the direction of increase of the K value; then, the envelope line *l* is rotated to find the critical point *T_B_* where *l* is tangent to the pig contour line, and the corresponding K value is obtained. The local contour *C*′ between the front foot point *T_A_* and the critical point *T_B_* of the point cloud C in the z coordinate is obtained by means of pass-through filtering. Then, the envelope line *l* is progressively translated to the right, and the last intersection point between the envelope line *l* and the local contour line *C*′ is identified as the heart girth measurement point *B*_1_.

#### 2.4.2. Shortest Path Method for Measuring Heart Girth

The heart girth point cloud *P_B_* is obtained by taking the heart girth measurement point *B*_1_ as the starting point and intercepting the circumferential perpendicular to the ground from the pose-normalized pig point cloud. Due to the limited perspectives of the sensors used to acquire the depth data, only points from the back and one side of the body of the pig are available; therefore, the obtained heart girth point cloud *P_B_* accounts for approximately 3/4 of the complete heart girth region of the pig, as shown in Figure 5c. Although a pig is a nonrigid object, the body of a pig in the individual information collection channel is relatively straight, and a specific part of its body can be approximated as a rigid object. A plane perpendicular to the ground through the *z*-axis is constructed, which is a plane of symmetry. Then, the method of mirror symmetry [41] is applied to obtain the complete heart girth point cloud $P_{B}^{\prime }$. As shown in Figure 5d, two points *B*_2_ and *B*_3_ are selected from the point cloud $P_{B}^{\prime }$ that form a triangle with the heart girth measurement point *B*_1_. Considering that the heart girth region of a pig is an irregular circle, to ensure the formation of a closed contour when calculating the shortest path, we look for two measurement points *B*_2_ and *B*_3_ at a distance of approximately 10 cm from the *z*-axis on both sides to ensure that there is a certain fault tolerance between these two measurement points. Then, the distances along the curve of the point cloud between pairs of adjacent points *B*_1_ and *B*_2_, *B*_2_ and *B*_3_ and *B*_3_ and *B*_1_ are calculated, and their sum is taken as the heart girth. When the distance between two points in a point cloud is sufficiently small, the distance along the curve between them can be approximated as the shortest distance between these two points. The classic algorithm for calculating the shortest distance between two points is the Dijkstra algorithm [42]. This algorithm converts the structure of a point cloud into a graph structure in the form of an undirected weighted adjacency table. The vertices adjacent to each vertex are obtained using the K-nearest neighbour search method, and these adjacent vertices are connected to form edges. The edge weights are calculated as the Euclidean distances between each vertex and its adjacent vertices. Taking the calculation of the curve distance between *B*_1_ and *B*_2_ as an example, the specific steps are as follows:

(a) Let *B*_1_ be the source point of the graph, let *C*(*B*_1_) = 0 and let *C*(*x_i_*) = ∞, where *C*(*B*_1_) is the weight from *B*_1_ to itself and *C*(*x_i_*) is the weight from the current *B*_1_ to other nodes. Define *S*(*x_i_*) as the set of points that have not yet been visited.
(b) Traverse *S*(*x_i_*) to find the node $x_{1}$ with the minimum weight *C*(*x_i_*) with respect to *B*_1_. Node *x*_1_ becomes the current node, and *x*_1_ is removed from *S*(*x_i_*).
(c) Compare the current node with each of its adjacent nodes *x*_2_ in *S*(*x_i_*) as follows: if *C*(*x*_1_) + *W*(*x*_1_,*x*_2_) < *C*(*x*^2^), then the path from *x*_1_ to *x*_2_ is *B*_1_ → *x*_1_ → *x*_2_. Remove *x*_2_ from *S*(*x_i_*).
(d) Repeat steps b and c until the current node is *B*_2_; then, *C*(*B*_2_) is the curve distance between *B*_1_ and *B*_2_.

## 3. Results and Discussion

### 3.1. Comparison of Two-view and Single-view Data Acquisition Result

**Figure 6:**
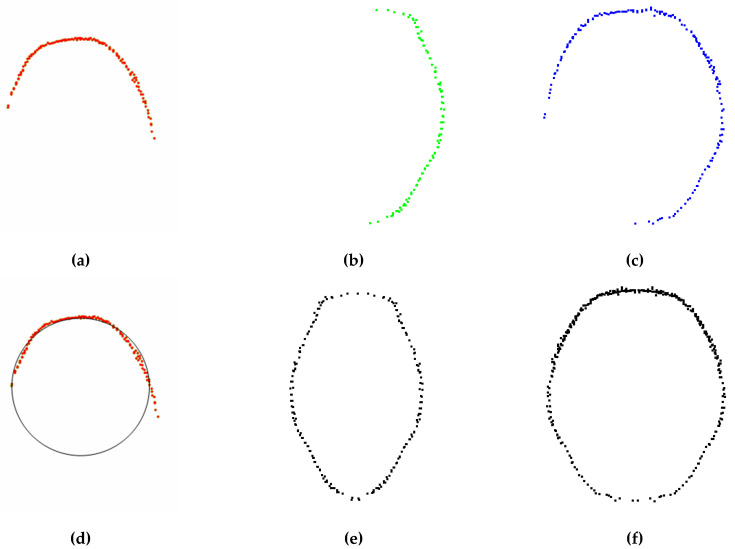
The first line is the heart girth point cloud obtained by different data acquisition ways. The second line is the complete heart girth point cloud obtained by different data acquisition ways. (**a**) Pig heart girth point clouds obtained by a top-view Kinect sensor; (**b**) Pig heart girth point cloud obtained by a side-view Kinect sensor; (**c**) Pig heart girth point cloud obtained by two-view Kinect sensors; (**d**) Fitting heart girth point cloud based on top-view point cloud; (**e**) Pig heart girth point cloud obtained by mirror symmetry based on side-view point cloud; (**f**) Pig heart girth point cloud obtained by mirror symmetry based on two-view point cloud.

In order to verify the advantages of the two-view Kinect sensors synchronous data acquisition method described in this paper, a pig was randomly selected to use a top-view Kinect sensor, a side-view Kinect sensor, and two-view Kinect sensors to collect pig point clouds and measure heart girth. Figure 6a–c are heart girth point clouds obtained by a top-view Kinect sensor, a side view Kinect sensor, and two-view Kinect sensors, respectively. As a top-view Kinect sensor can only collect the pig back point cloud, the method proposed in this paper cannot locate the heart girth position and measure heart girth. We used the method in the [43] to measure heart girth based on top-view point cloud, and the fitting heart girth point cloud is shown in Figure 6d. When using proposed measurement method to measure heart girth based on side-view point cloud, the heart girth point cloud obtained by mirror symmetry is shown in Figure 6e. When using two-view Kinect sensors to synchronously collect data for heart girth measurement, the heart girth point cloud obtained by mirror symmetry is shown in Figure 6f. The measurement results of the above 3 heart girth point clouds are shown in Table 1. It can be seen from the table that the absolute error of heart girth measured by a top-view Kinect sensor and a side-view Kinect sensor respectively is large, but measured by two-view Kinect sensors is small. The reason is that it can be seen from Figure 6a,d that heart girth is measured only according to the top-view data. Due to the lack of abdomen, the shape of the fitting heart girth point cloud is greatly different from the actual heart girth. It can be seen from Figure 6b,e that when using only a side-view point cloud for heart girth measurement, the accurate back width cannot be obtained due to the lack of back data, and the measurement error is large. When using two-view Kinect sensors to measure heart girth (as shown in Figure 6c,f), we can obtain heart girth point cloud which accounts for approximately 3/4 of the complete heart girth. Through mirror symmetry, we can get complete heart girth point cloud, and the shape is close to the actual heart girth. 

**Figure 7:**
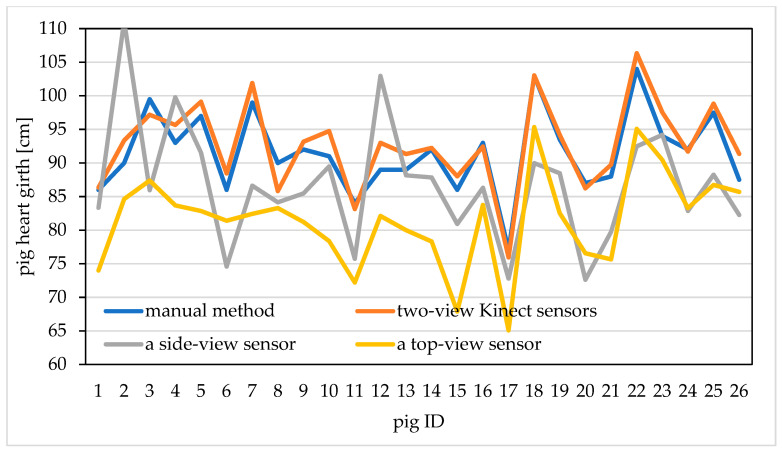
Comparison between the measured value obtain by different data acquisition methods and the manual value.

We used 26 pigs to compare the influence of three data acquisition ways on the measurement accuracy of heart girth. The heart girth point clouds obtained by three acquisition ways were measured, and the measurement results and manual heart girth value are shown in Figure 7. From this figure, we can see that the heart girth measured by using the two-view Kinect sensors synchronous acquisition is closer to the manual heart girth value, with smaller error, while the heart girth measured by using a side-view Kinect sensor and a top-view Kinect sensor has larger error. Therefore, this paper uses two-view Kinect sensors to synchronously collect pig point cloud, which provides data support for the realization of higher precision heart girth measurement.

### 3.2. Pig Point Cloud Registration Results

To verify the feasibility and effectiveness of the feature-based improved 4PCS registration method proposed in this paper, the proposed registration method and the traditional point cloud registration method (4PCS registration combined with ICP registration) were both used to register two-view depth images of six pigs with different body positions (standing, walking, looking up, bowing, and arching the back) and of different breeds (five Large White pigs and one Landrace pig). 

**Figure 8:**
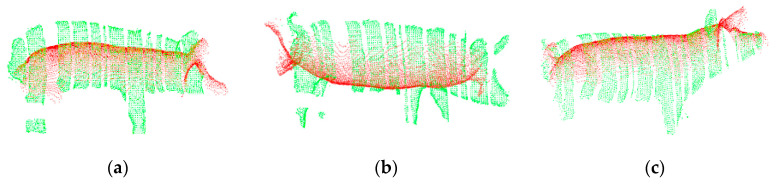

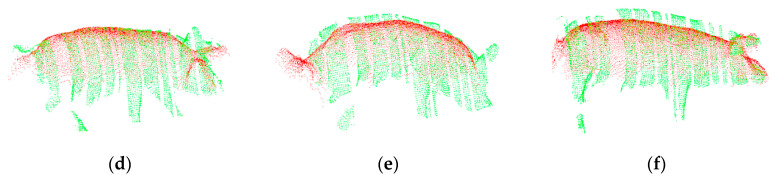
Registration results of the traditional point cloud registration method: (**a**) Large White pig in a standing pose; (**b**) Large White pig in a walking pose; (**c**) Large White pig in an upward-looking pose; (**d**) Large White pig in a bowing pose; (**e**) Large White pig with an arched back; (**f**) Landrace pig.

**Figure 9:**
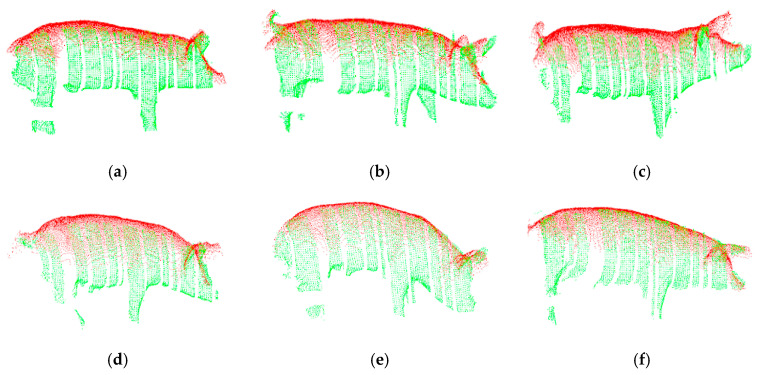
Registration results of the method proposed in this paper: (**a**) Large White pig in a standing pose; (**b**) Large White pig in a walking pose; (**c**) Large White pig in an upward-looking pose; (**d**) Large White pig in a bowing pose; (**e**) Large White pig with an arched back; (**f**) Landrace pig.

The registration results obtained using the traditional point cloud registration method are shown in Figure 8, and the registration results obtained using the proposed method are shown in Figure 9. In these figures, the top-view pig point cloud is shown in red, and the side-view pig point cloud is shown in green. From Figure 8, it can be seen that when the traditional point cloud registration method is used to register the two-view pig point clouds, the registration results may be rotated along the back; the registration of the back, ears, tail and other feature-rich parts may fail; and the heads and tails of the two point clouds may even point in opposite directions after registration. By contrast, it can be seen from Figure 9 that the point cloud registration results of the proposed method along the back, ears and tail are good regardless of the pig’s posture, and this method is applicable to pigs of different breeds.

**Figure 10:**
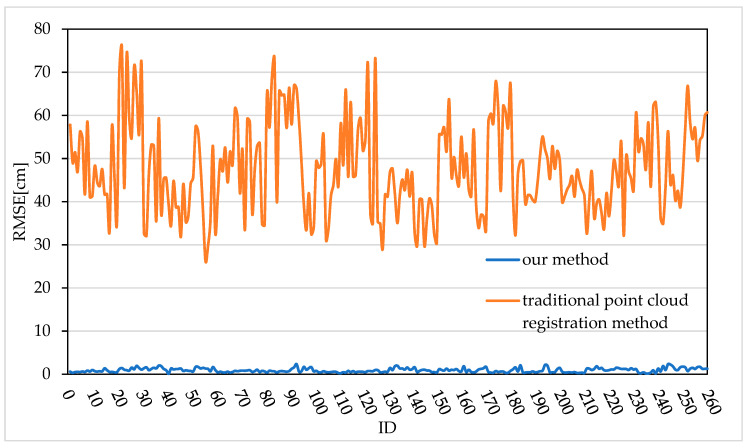
RMSEs of our method and the traditional point cloud registration method.

Ten frames of top-view images and corresponding side-view images of each of the 26 live pigs were selected. After the two-view depth image data of the 26 pigs were preprocessed, the method proposed in this paper and the traditional point cloud registration method were used for registration; in total, 260 live pig point cloud registration results were obtained. To further analyse the accuracy of the proposed registration method and the traditional point cloud registration method, the root mean square error (RMSE) is introduced to quantitatively evaluate the registration error of each of the 260 registration results, as shown in Figure 10. The RMSE was calculated as the average Euclidean distance between corresponding point pairs after registration. The smaller the RMSE value is, the smaller the registration error, and the better the registration effect. Ideally, when the point clouds are fully correctly registered, the average Euclidean distance between corresponding point pairs will be 0. Figure 10 shows that the registration error of the traditional point cloud registration method fluctuates between 26.25 cm and 75.94 cm, while the registration error of the method proposed in this paper ranges from 0.10 cm to 2.34 cm. Thus, the registration error of the proposed method is obviously smaller than that of the traditional point cloud registration method, indicating that the registration accuracy of the proposed method is higher. In summary, this paper proposes a feature-based improved 4PCS registration method for two-view pig point cloud in which the feature-rich ear and tail regions and the back contour region of the side-view point cloud are extracted to serve as the registration regions, thereby effectively reducing the matching error and improving the registration accuracy.

In order to further verify the influence of the proposed registration method on the subsequent improvement of the heart girth measurement accuracy, a comparative test was conducted on the heart girth measurement accuracy of 26 pig point clouds after registration with the proposed registration method and the traditional point cloud registration method.

**Figure 11:**
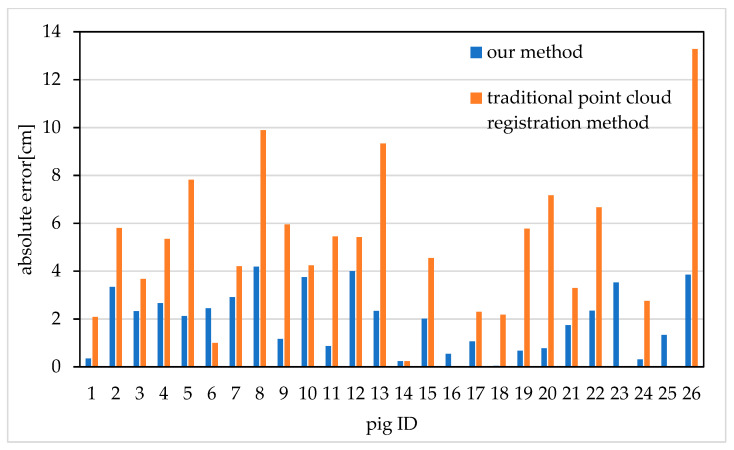
Absolute errors of pig heart girth measurements after using different registration methods.

The results are shown in Figure 11. It should be noted that because of the obvious failure of the traditional point cloud registration method, we remove the failed data before the heart girth measurement. The traditional point cloud registration methods fail to register pig 16, pig 23 and pig 25, so it is impossible to calculate the heart girth and error. The average absolute error of heart girth measurement is 5.15 cm after point cloud registration with traditional point cloud registration method. The average absolute error of heart girth measurement is 1.96 cm after using the proposed feature-based improved 4PCS registration method. Compared with the traditional point cloud registration method, the average absolute error of heart girth measurement is reduced by 3.19 cm. Therefore, the proposed feature-based improved 4PCS registration method provides data support for the subsequent improvement of heart girth measurement accuracy.

### 3.3. Pig Heart Girth Measurement Results 

#### 3.3.1. Results of Pig Heart Girth Measurement Point Extraction

To verify the validity of the proposed heart girth measurement point extraction method, the LSSA-CAU software was used to interactively select the heart girth measurement points from the pose-normalized pig point clouds. The selected results were compared with the coordinate values extracted using the method proposed in this paper. To reduce the error of interactive point selection using the LSSA-CAU software, each measurement point was selected three times, and the average value was calculated for use in the comparison. The heart girth measurement takes the measurement point as the starting point and intercepts the circumferential perpendicular to the ground from the pose-normalized pig point cloud. Therefore, the z coordinate is the same for all points in the heart girth point cloud, so only the z coordinate needs to be identified to extract the heart girth measurement point. Consequently, when verifying the accuracy of heart girth measurement point extraction, only the error in the *z*-axis direction between the actual and measured points needs to be calculated.

**Figure 12:**
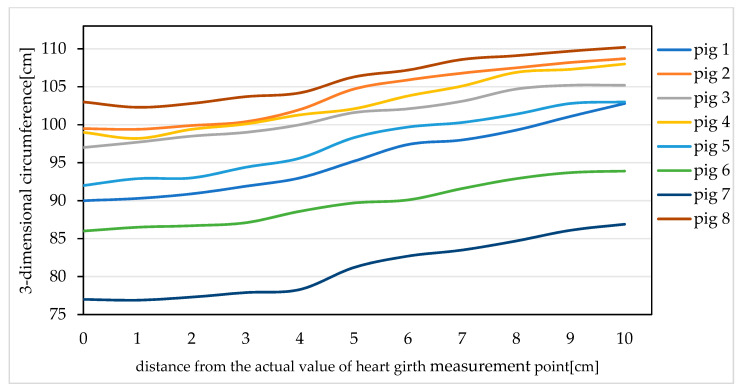
Curves representing the 3-dimensional circumferences of individual pigs starting from the actual heart girth measurement point and moving towards the hips (the origin of the horizontal coordinate corresponds to the actual heart girth measurement point, and the value on the vertical axis corresponding to the origin is the true heart girth value of the pig).

We selected eight pigs and analysed the three-dimensional circumference values of each pig within 10 cm towards the hip starting from the actual heart girth measurement point; the results are shown in Figure 12. It is found that within 4 cm of the actual heart girth measurement point, the three-dimensional circumference of each pig is fairly constant. Therefore, within this range, the three-dimensional circumference value is reasonably close to the actual heart girth value, while beyond 4 cm, the curves of the three-dimensional circumference values of the pigs are relatively steep, meaning that the three-dimensional circumferences in this range are quite different from the actual heart girth value. From the above analysis, it can be seen that if the distance between the actual heart girth measurement point and the extracted point is greater than 4 cm, the error between the heart girth value measured starting from the extracted heart girth measurement point and the actual heart girth value will be large, thus affecting the accuracy of the heart girth measurement. Therefore, in this paper, when the error between the actual heart girth measurement point and the point extracted using the proposed method is less than or equal to 4 cm, the heart girth measurement point is considered to be extracted successfully. Otherwise, the extraction of the heart girth measurement point is considered to have failed.

**Figure 13:**
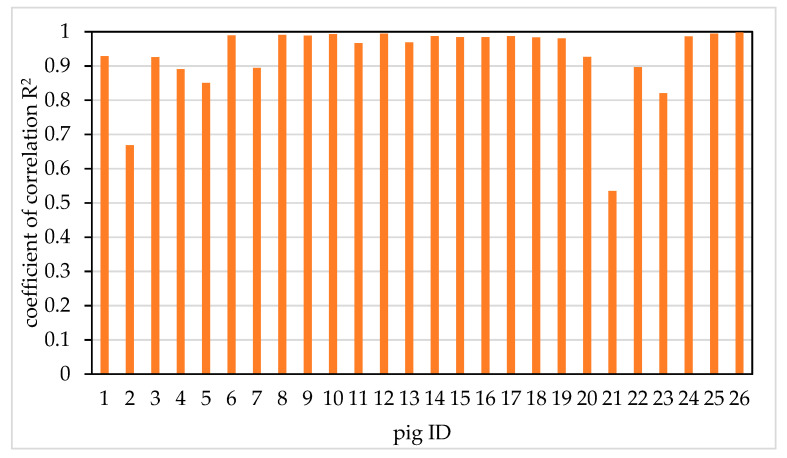
The coefficient of correlation R^2^ between the actual value and the measured value of each pig’s heart girth measurement point R^2^ of 26 pigs.

Extracting the heart girth measurement points of 26 pigs. It is worth noting that each pig has 10 frames, so we need to extract heart girth measurement points from 10 frames, respectively. Then the linear analysis of the actual value and the measurement value of the heart girth measurement point of each pig was carried out. The coefficient of determination R^2^ between the actual value and the measured value of each pig’s heart girth measurement point was calculated. The coefficient of correlation R^2^ of 26 pigs is shown in Figure 13. It can be seen from the figure that the R^2^ of pig 2 and pig 21 is smaller. The reason is that there is one frame of heart girth measurement point extraction failure in Pig 2, the absolute error between the actual value and the measured value of heart girth measurement point is 5.27 cm, while there are three frames of heart girth measurement point extraction failure in pig 21, the absolute error is 5.10, 5.34 and 5.20 cm, respectively. The R^2^ of the other 24 pigs were all above 0.82. The results showed that proposed method can accurately extract heart girth measurement point. The error of heart girth measurement point extraction method basically meets the demand of heart girth measurement.

To further verify the influence of pig posture on the point extraction results, all 260 pig point clouds registration results were analysed; 166 of the pigs were found to be in a natural standing state, and the remaining 94 pigs were in a walking state. A comparative test was carried out on the results of heart girth measurement point extraction for pigs in the standing state and the walking state, and the extraction success rate and average absolute error were calculated separately. The test results are shown in Table 2. The successful extraction rate for pigs in the standing state is 97.6%, and that for pigs in the walking state is 95.7%. The results indicate that the success rate of heart girth measurement point extraction is slightly lower for pigs in the walking state than for pigs in the standing state. From an analysis of the failure cases, the reason appears to be that when a pig is walking forward with the leg joint of the front leg on the side close to the side-view sensor is bent backwards at a large angle, the proposed point extraction method will fail; however, this is an instantaneous movement state that will rarely be captured by the side-view sensor. The average absolute error of detecting the heart girth measurement points of pigs in both the standing and walking states is less than 4 cm, which is sufficient to meet the needs of heart girth measurement. Therefore, the method proposed in this paper is virtually independent of the pig posture and can be used to accurately extract the heart girth measurement points of pigs. It can provide possibility for the subsequent improvement of heart girth measurement accuracy.

#### 3.3.2. Accuracy of Pig Heart Girth Measurement

**Figure 14:**
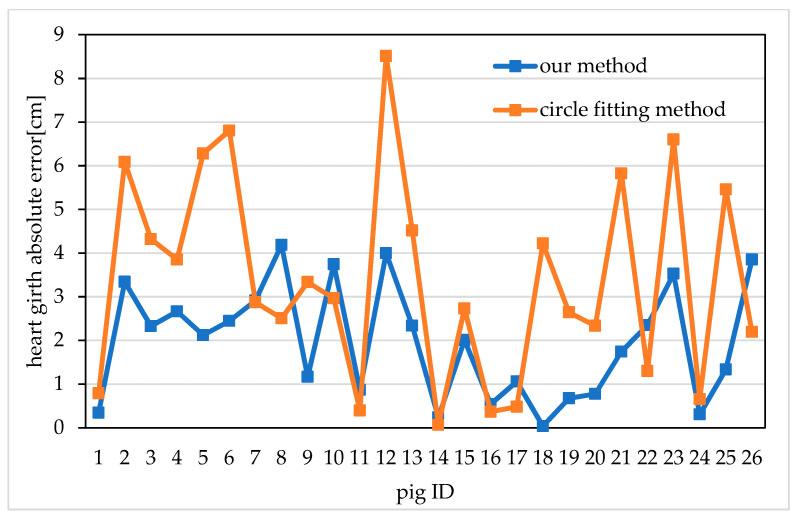
Absolute errors of pig heart girth measurements obtained using different methods.

To verify the accuracy of the proposed heart girth measurement method, the 26 pigs were measured manually, using the circle fitting method [43], and using the shortest path method proposed in this paper. During the image acquisition period, professional stockmen were also asked to measure the pigs’ heart girths manually using a tape measure during feeding. Because manual measurements will be affected by the measurement position, the measurement force and the activity of the pigs, to reduce the error of the manual measurements, the average value of three manual measurements was taken as the actual manual measurement value for each pig, and the measurement accuracy was found to be 1 mm. Then, the proposed method and the circle fitting method were used to measure the heart girth of 10 frames heart girth point cloud for each pig, and respectively obtained 10 sets of measurements of heart girth for each pig. The average value of the 10 sets of measurements was taken as the measured heart girth measurement of this pig. It should be emphasized that if there are a few frames of this pig that fails to extract the heart girth measurement point, it needs to be removed, and then the remaining frames are measured, and the average value is taken as the measured heart girth measurement of this pig. Finally, the absolute and relative errors between the values measured using these two methods and the actual manually measured values were calculated. The absolute errors of the heart girth measurements for the 26 pigs are shown in Figure 14. 

From Figure 14 we can see that the absolute errors obtained using the proposed method are all less than 4.19 cm, and the results are stable. In contrast, the absolute errors obtained using the circle fitting method are as large as 8.51 cm, and the measurement results show relatively large fluctuations. The average absolute errors and relative errors of heart girth measurement for the 26 pigs were also calculated. The average absolute error achieved using the proposed method is 1.96 cm, and the average relative error is 2.14%; by contrast, the average absolute error achieved using the circle fitting method is 3.39 cm, and the average relative error is 3.68%. The above data show that the heart girth values measured using the proposed method are more accurate and stable than those measured using the circle fitting method. The proposed method effectively improves the measurement accuracy of pig heart girth.

**Figure 15:**
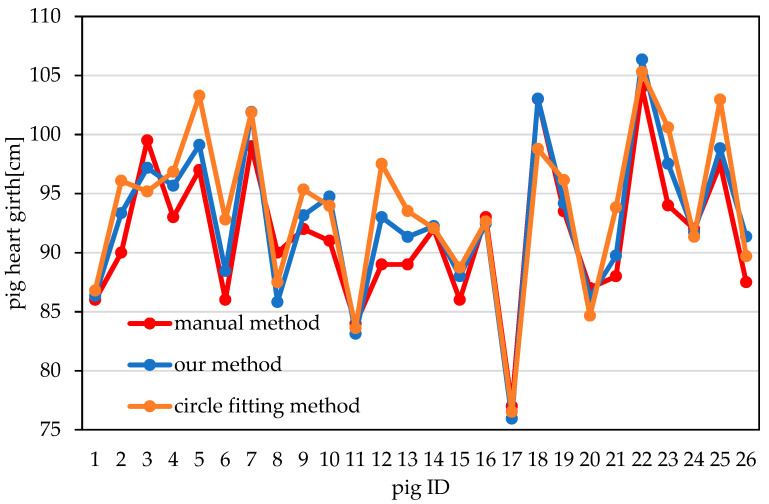
Pig heart girth measurements obtained using different methods.

The actual heart girth values that were measured manually for the 26 pigs and the values obtained using the two automated methods are shown in Figure 15. From Figure 15, it can be seen that the heart girths obtained using the automated methods are sometimes larger and sometimes smaller than those obtained through manual measurement. The reason is that only point cloud data from the back and one side of the pigs were collected in this experiment, while point cloud data from the other side were missing. Since the two sides of a pig are not completely symmetrical, the estimation of the point cloud on the missing side from the existing point cloud data that was performed during the automated measurement procedure could cause the final measured heart girth value to be either larger or smaller than the actual value.

## 4. Conclusions

In this study, we propose a new non-contact method of measuring pig heart girth using two Kinect sensors. Our method can register the two-view point clouds, extract the measurement point, obtain the complete heart girth point cloud by mirror symmetry, and calculate the heart girth length. This method has been verified by 26 live pigs in the farm. The feature-based improved 4PCS registration method provides data support for the realization of higher precision heart girth measurement. The results of heart girth measurement point extraction can basically meet the needs of heart girth measurement. The average relative error of heart girth measurement was 2.14%, which effectively improves the measurement accuracy of the pig heart girth and obtains satisfactory results. 

However, the accuracy of the measurement points extraction needs to be further improved in our future work. At the same time, the proposed heart girth measurement method is used to verify more pigs, which is expected to be integrated into the software system and applied to commercial farms.

## Figures and Tables

**Table 1:** Pig heart girth measurement results obtain by different data acquisition ways.

Data Acquisition Method	Heart Girth/cm	Absolute Error/cm
a top-view Kinect sensor	87.38	12.12
a side-view Kinect sensor	85.95	13.54
two-view Kinect sensors	97.17	2.33

**Table 2:** Results of heart girth measurement point extraction for pigs in different states.

Pig Posture	Pig Point Cloud Parameters			
	Number of Samples	Number of Successful Extractions	Successful Extraction Rate/%	Mean Absolute Error/cm
standing	166	162	97.6%	1.76
walking	94	90	95.7%	2.03

## Appendix A

The method of extracting the contour of the pig point cloud is as follows:

(a) Project the point *P_i_*(*x,y,z*) and its K-neighbourhood point set *N*(*P_i_*) onto the least-squares plane *I* to obtain the projection points $P_{i}^{\prime }$ and $N_{j}^{\prime }\left(j=0,1,\ldots ,K-1\right)$.
(b) Starting from $P_{i}^{\prime }$, connect $P_{i}^{\prime }$ to each $N_{j}^{\prime }\left(j=0,1,\ldots ,K-1\right)$ to obtain the projection vectors $P_{i}^{\prime }N_{j}^{\prime }\left(j=0,1,\ldots ,K-1\right)$.
(c) Select any projection vector as a reference and calculate the angles *α_j_* between it and the other projection vectors.
(d) Calculate the angles *β_j_* between the other projection vectors and the normal vector ${\vec{n}}_{i}$. If *β_j_* ≥ 90°, then:
  (A1) $$\alpha_{j}=360^{\circ }-\alpha_{j}$$
(e) Sort the angle sequence *θ* = (α_0_,α_1_,…,α*_K_*_−1_) in ascending order and add the two angle values 0 and 2π to obtain the new angle sequence *θ’* = (0,α_0_,α_1_,…,α*_K_*_–1_,2*π*). Then, calculate the angles *δ_j_* between adjacent projection vectors:
  (A2) $$\delta_{j}=\theta_{j+1}-\theta_{j}\left(j=0,1,\ldots K-1\right)$$
  and judge whether *δ*_max_ is greater than a threshold *ε*. If *δ*_max_ ≥ *ε*, the current point is marked as a boundary point; otherwise, it is not a boundary point.
(f) Loop through (a) to (e) until all points have been traversed. All identified boundary points form the pig contour.

## References

[1] Klingenberg C.P. (2010). Evolution and development of shape: Integrating quantitative approaches. Nat. Rev. Genet..

[2] Wang Y., Yang W., Walker L., Rababah T. (2008). Enhancing the accuracy of area extraction in machine vision-based pig weighing through edge detection. Int. J. Agric. Biol. Eng..

[3] Machebe N., Ezekwe A., Okeke G., Banik S. (2016). Path analysis of body weight in grower and finisher pigs. Indian J. Anim. Res..

[4] Pezzuolo A., Guarino M., Sartori L., González L.A., Marinello F. (2018). On-barn pig weight estimation based on body measurements by a Kinect v1 depth camera. Comput. Electron. Agric..

[5] Schofield C.P., Marchant J.A., White R.P., Brandl N., Wilson M. (1999). Monitoring pig growth using a prototype imaging system. J. Agric. Eng. Res..

[6] Augspurger N.R., Ellis M. (2002). Weighing affects short-term feeding patterns of growing-finishing pigs. Can. J. Anim. Sci..

[7] Pezzuolo A., Milani V., Zhu D., Guo H., Guercini S., Marinello F. (2018). On-barn pig weight estimation based on body measurements by structure-from-motion (SfM). Sensors.

[8] Huang L., Li S., Zhu A., Fan X., Zhang C., Wang H. (2018). Non-contact body measurement for qinchuan cattle with LiDAR sensor. Sensors.

[9] Chan T.O., Lichti D.D., Jahraus A., Esfandiari H., Lahamy H., Steward J., Glanzer M. (2018). An egg volume measurement system based on the microsoft kinect. Sensors.

[10] Vázquez-Arellano M., Griepentrog H.W., Reiser D., Paraforos D.S. (2016). 3-D imaging systems for agricultural applications—a review. Sensors.

[11] Sansoni G., Trebeschi M., Docchio F. (2009). State-of-the-art and applications of 3D imaging sensors in industry, cultural heritage, medicine, and criminal investigation. Sensors.

[12] Tasdemir S., Urkmez A., Inal S. (2011). Determination of body measurements on the Holstein cows using digital image analysis and estimation of live weight with regression analysis. Comput. Electron. Agric..

[13] Azzaro G., Caccamo M., Ferguson J.D., Battiato S., Farinella G.M., Guarnera G.C., Puglisi G., Petriglieri R., Licitra G. (2011). Objective estimation of body condition score by modeling cow body shape from digital images. J. Dairy Sci..

[14] Mortensen A.K., Lisouski P., Ahrendt P. (2016). Weight prediction of broiler chickens using 3D computer vision. Comput. Electron. Agric..

[15] Shi C., Teng G., Li Z. (2016). An approach of pig weight estimation using binocular stereo system based on LabVIEW. Comput. Electron. Agric..

[16] Andújar D., Dorado J., Fernández-Quintanilla C., Ribeiro A. (2016). An approach to the use of depth cameras for weed volume estimation. Sensors.

[17] Kim J., Chung Y., Choi Y., Sa J., Kim H., Chung Y., Park D., Kim H. (2017). Depth-based detection of standing-pigs in moving noise environments. Sensors.

[18] Guo H., Ma X., Ma Q., Wang K., Su W., Zhu D. (2017). LSSA_CAU: An interactive 3d point clouds analysis software for body measurement of livestock with similar forms of cows or pigs. Comput. Electron. Agric..

[19] Salau J., Haas J., Junge W., Harms J., Bauer U., Suhr O., Bieletzki S., Schönrock K., Rothfuß H. (2018). An automated system to monitor dairy cows body condition using a time-of-flilght camera. Comput. Ind..

[20] Spoliansky R., Edan Y., Parmet Y., Halachmi I. (2016). Development of automatic body condition scoring using a low-cost 3-dimensional Kinect camera. J. Dairy Sci..

[21] Kongsro J. (2014). Estimation of pig weight using a Microsoft Kinect prototype imaging system. Comput. Electron. Agric..

[22] Condotta I.C.F.S., Brown-Brandl T.M., Silva-Miranda K.O., Stinn J.P. (2018). Evaluation of a depth sensor for mass estimation of growing and finishing pigs. Biosyst. Eng..

[23] Wang K., Guo H., Ma Q., Su W., Chen L., Zhu D. (2018). A portable and automatic Xtion-based measurement system for pig body size. Comput. Electron. Agric..

[24] Salau J., Haas J.H., Thaller G., Leisen M., Junge W. (2016). Developing a multi-Kinect-system for monitoring in dairy cows: Object recognition and surface analysis using wavelets. Animal.

[25] Salau J., Haas J.H., Junge W., Thaller G. (2017). A multi-Kinect cow scanning system: Calculating linear traits from manually marked recordings of Holstein-Friesian dairy cows. Biosyst. Eng..

[26] Salau J., Haas J.H., Junge W., Thaller G. (2017). Automated calculation of udder depth and rear leg angle in Holstein-Friesian cows using a multi-Kinect cow scanning system. Biosyst. Eng..

[27] Zhang H., Wei Q., Jiang Z. (2017). 3D reconstruction of space objects from multi-views by a visible sensor. Sensors.

[28] Pezzuolo A., Guarino M., Sartori L., Marinello F. (2018). A feasibility study on the use of a structured light depth-camera for three-dimensional body measurements of dairy cows in free-stall barns. Sensors.

[29] Wang K., Zhu D., Guo H., Ma Q., Su W., Su Y. (2019). Automated calculation of heart girth measurement in pigs using body surface point clouds. Comput. Electron. Agric..

[30] Godbehere A., Matsukawa A., Goldberg K. (2012). Visual tracking of human visitors under variable-lighting conditions for a responsive audio art installation. 2012 American Control Conference (ACC).

[31] Zhan Q., Yu L. (2012). Segmentation of LiDAR point cloud based on similarity measures in multi-dimension euclidean space. Adv. Intell. Soft Comput..

[32] Besl P.J., McKay N.D. (1992). A method for registration of 3-D shapes. IEEE Trans. Pattern Anal. Mach. Intell..

[33] He Y., Liang B., Yang J., Li S., He J. (2017). An iterative closest points algorithm for registration of 3D laser scanner point clouds with geometric features. Sensors.

[34] Aiger D., Mitra N.J., Cohen-Or D. (2008). 4-points congruent sets for robust pairwise surface registration. ACM Trans. Graph..

[35] Li P., Wang R., Wang Y., Gao G. (2020). Fast Method of Registration for 3D RGB Point Cloud with Improved Four Initial Point Pairs Algorithm. Sensors.

[36] Zhang S., Wang H., Gao J.G., Xing C.Q. (2019). Frequency domain point cloud registration based on the Fourier transform. J. Vis. Communi. Image Represent..

[37] Huang J.H., Wang Z., Gao J.M., Huang Y.P., Towers D.P. (2017). High-Precision Registration of Point Clouds Based on Sphere Feature Constraints. Sensors.

[38] Papadakis P., Pratikakis I., Perantonis S., Theoharis T. (2007). Efficient 3D shape matching and retrieval using a concrete radialized spherical projection representation. Pattern Recognit..

[39] Juyoung Y., Cheng S.-W., Cheong O., Vigneron A. (2017). Finding largest common point sets. Int. J. Comput. Geom. Appl..

[40] Groesbeck C.N., Lawrence K.R., Young M.G., Goodband R.D., DeRouchey J.M., Tokach M.D., Nelssen J.L., Dritz S.S. (2002). Using heart girth to determine weight in finishing pigs. Kans. Agric. Exp. Stn. Res. Rep..

[41] Guo H., Li Z., Ma Q., Zhu D., Su W., Wang K., Marinello F. (2019). A bilateral symmetry based pose normalization framework applied to livestock body measurement in point clouds. Comput. Electron. Agric..

[42] Möhring R.H., Schilling H., Schütz B., Wagner D., Willhalm T., Nikoletseas S.E. (2005). Partitioning graphs to speed up Dijkstra’s algorithm. Experimental and Efficient Algorithms. WEA 2005. Lecture Notes in Computer Science.

[43] YOSHIDA K., KAWASUE K. Robust 3D Pig Measurement in Pig Farm. Proceedings of the European Conference on Computer Vision (ECCV).

